# Supplementary material for: Are carbon emissions trading and green financial instruments synergistic? -Comprehensive quantitative research based on content analysis
Source: PLoS One. 2024 Mar 7;19(3):e0298601. doi: 10.1371/journal.pone.0298601 (PMC10919601; doi:10.1371/journal.pone.0298601)
Supplement: S1 Data — (ZIP) [file pone.0298601.s001.zip › Data/地级市Z_F工作报告.zip!/╜¡╬≈/╔╧╚─╩╨/└┤╫╘╣1⁄2╓┌║┼í╛┬φ┐╦╩2╛▌═°í┐_2011.docx]

各位代表：

现在，我代表市人民政府，向大会报告政府工作，并就《上饶市国民经济和社会发展第十二个五年规划纲要 ( 草案 ) 》作出说明，请予审议，并请市政协各位委员和列席会议的同志提出意见。

一、 “ 十一五 ” 时期的主要成效和 2010 年的工作回顾

“ 十一五 ” 时期，是我市经济社会发展最好最快的五年之一。五年来，在省委、省政府和市委的正确领导下，在市人大、市政协的监督支持下，全市人民励精图治、奋发有为，成功战胜历史罕见的低温雨雪冰冻灾害、特大洪涝灾害，科学应对国际金融危机的严重冲击，全面完成 “ 十一五 ” 规划确定的各项目标任务，经济社会发展取得了重大成就，呈现出八个方面的显著成效：

一是全市生产总值实现翻番，财政总收入突破百亿元大关，综合实力明显增强。 与 “ 十五 ” 期末相比，全市生产总值由 388.1 亿元增加到 901 亿元，完成 “ 十一五 ” 规划目标的 107.9% ，年均增长 13.8% （可比价）。财政总收入由 33.6 亿元增加到 113.9 亿元，完成 “ 十一五 ” 规划目标的 153.9% ，年均增长 27.7% 。三次产业结构由 21.2:42.4:36.4 调整为 16.8:51:32.2 。

二是规模以上工业主营业务收入突破千亿元，工业增加值占生产总值的比重提高 11 个百分点，主攻工业成效显著。 全市规模以上工业增加值由 “ 十五 ” 期末的 53.3 亿元增加到 266.2 亿元，年均增长 37.9% （现价）。工业增加值占生产总值的比重由 “ 十五 ” 期末的 30.9% 提高到 42.1% 。上饶经济技术开发区升格为国家级开发区，这是上饶工业发展跃上新台阶的重要标志。

三是三清山、龟峰相继 “ 申遗 ” 成功，旅游接待人数和综合收入双双跃居全省第一位，旅游产业强势突起。 接待国内外游客人数由 “ 十五 ” 期末的 570 万人次增加到 1986.8 万人次；实现旅游综合收入由 “ 十五 ” 期末的 32.5 亿元增加到 141.6 亿元，占生产总值的比重达到 16.7% 。

四是全市城镇化率年均提高 2 个多百分点，城市建成区面积达到 212 平方公里，城乡面貌深刻变化。 全市城市建成区面积比 “ 十五 ” 期末扩大了 80.6 平方公里，城镇化率有望由 “ 十五 ” 期末的 27% 提高到 40% ；市中心城区面积达到 55.8 平方公里，比 “ 十五 ” 期末扩大了 20.6 平方公里；人口达到 52.7 万人，比 “ 十五 ” 期末增加了 17.7 万人。五年来，全市共投入新农村建设资金 37.4 亿元，完成 6570 个村点建设，近三分之一的农民基本实现 “ 走上平坦路、喝上干净水、用上卫生厕、洗上热水澡、用上洁净灶 ” 的目标。

五是粮食产量持续突破 60 亿斤，农民人均纯收入突破 5000 元， “ 三农 ” 基础更加牢固。 与 “ 十五 ” 期末比较，粮食总产由 52.4 亿斤增加到 61 亿斤，连年创历史新高；农民人均纯收入由 “ 十五 ” 期末的 3226 元增加到 5317 元，年均增长 10.5% ，增幅较 “ 十五 ” 期间加快 0.9 个百分点。

六是高速公路、高速铁路的 “ 双十字 ” 构架加快建设，高速公路总里程全省第一，交通枢纽初步成型。 全市公路总里程达到 1.8万公里，高速公路总里程达到382.1 公里。德昌、德上、上武高速公路加快建设；尤其是合福、杭南长两条高铁的开工建设，为上饶成为我国重要的交通枢纽奠定了基础。

七是海关、无水港等开放口岸和平台相继建成，外贸出口首次突破 15 亿美元，开放水平不断提升。 开放城市获得国务院批准，被国家商务部授予国家加工贸易重点承接地，被国台办和省政府授予台商企业重点承接地。对外交流实现突破。全市外贸进出口由 “ 十五 ” 期末的 1.47 亿美元增加到 16.7 亿美元，是 “ 十五 ” 期末的 11.4 倍，年均增长 62.5% 。

八是节能减排措施进一步落实，安全稳定形势进一步好转，各项保障体系进一步完善，社会大局和谐稳定。 全市 森林覆盖率提高 4 个百分点、达到 61.7% 。超额完成 “ 十一五 ” 减排任务。 财政投入民生资金 136.5 亿元，办好了八个方面的实事。 “ 十一五 ” 期间，全市未发生重大安全生产责任事故、重大群体性事件和重大治安灾害事故。教育、科技、文化、卫生、体育等事业稳步发展，社会治安稳定，人民生活水平不断提高，幸福指数不断提升。

2010 年是 “ 十一五 ” 时期收官之年，也是上饶撤地设市十周年。我们围绕市二届人大六次会议确定的各项目标任务，坚定不移地实施重大项目带动战略，加快融入鄱阳湖生态经济区建设，不断深化改革开放，着力保障和改善民生，经济社会保持平稳较快的发展势头。全市生产总值同比增长 14.8% 。财政总收入同比增长 36.4% ，其中一般预算收入达到 72.6 亿元，同比增长 50.9% ；各县（市、区）财政总收入均超过 4 亿元，有 10 个县（市、区）财政总收入超 5 亿元，其中铅山县、信州区财政总收入增幅超过 50% 。规模以上工业增加值同比增长 22.6% 。固定资产投资达到 805.4 亿元，同比增长 33.6% 。社会消费品零售总额达到 330.5 亿元，同比增长 19.6% 。城镇居民人均可支配收入达到 15535 元，同比增长 11% ；农民人均纯收入同比增长 13.1% 。

一年来，我们主要做了以下六个方面的工作：

（一） “ 三化 ” 联动，增强了经济发展的支撑力

牢牢抓住鄱阳湖生态经济区上升为国家战略的历史机遇，全力推进新型工业化、新型城镇化、农业现代化，促进了速度、结构、效益相协调。

新型工业化加速推进。 全市规模以上工业主营业务收入达到 1177.2 亿元，利税达到 122.9 亿元。工业园区集聚效应进一步显现，全市工业园区完成开发面积 63.5 平方公里，实现主营业务收入 1136 亿元，同比增长 47% ；实现税金 63.5 亿元，同比增长 58.7% ；主营业务收入超百亿元的园区达到 4 个，其中上饶经济技术开发区达到 284 亿元、广丰工业园区达到 226 亿元，横峰和玉山工业园区分别达到 135 亿元和 101 亿元。主导产业支撑作用进一步显现。四大主导产业实现主营业务收入 863 亿元，同比增长 51% ，占全市规模以上工业比重达到 74% ，其中新能源产业占全市规模以上工业比重达到 13.3% 。工业发展后劲进一步显现。全年完成工业投资 383 亿元，占全部固定资产投资的 47.6% 。晶科能源电池组件、德铜富家坞扩建、和丰铜业、富旺铜业、海螺水泥、索普信节能灯等一批重大项目顺利推进。全市主营业务收入过亿元的工业企业达到 230 户，其中过 50 亿元企业实现零的突破。

新型城镇化加力提升。 坚持以城带乡、统筹发展，努力形成中心城市、县城、重点镇、中心村多层推进、多点支撑的新型城镇化发展格局。 全市开工建设城建项目 520 个，完成投资 386.7 亿元，其中市中心城区开工建设重点项目 32 个，完成投资 90.2 亿元。城市重点工程加快推进，功能不断完善，品位持续提升。中心城区被评为全省双拥模范城、省级卫生城市、省级园林城市。新农村建设成效显著，完成了 1319 个新农村村点建设任务，湖区生态渔村、景区旅游新村、城郊社区新村特色进一步彰显。

农业现代化加快步伐。 全面落实各项强农惠农政策，通过 “ 一卡通 ” 发放各项惠农补贴 20 亿元。深入开展农业高产创建活动，促进了主要农产品增产增收。鄱阳县被授予 “ 全国粮食生产先进县标兵 ” 称号。农业产业化进程加快，省级以上农业龙头企业 70 家，数量继续位居全省首位； 192 家市级以上龙头企业实现销售收入 165 亿元；农产品出口达 3.5 亿美元，首次跃居全省第一。尤其是面对历史罕见的特大洪涝灾害，全市上下万众一心，众志成城，创下了 “ 未垮一坝、未倒一堤 ” 的奇迹，夺取了抗洪斗争的全面胜利。

（二）消费拉动，增强了现代服务业的竞争力

旅游产业快速壮大。 三清山被国家旅游局列为 5A 级旅游景区预备名录。重点景区配套设施进一步完善。婺源县成为全国首批 5 个旅游标准化试点县之一， 玉山县成功创建全省旅游强县。全市有世界自然遗产地 2 处，国家级风景名胜区 3 处，国家湿地公园 1 处，国家 4A 级景区 10 处；全年新增农家乐 8274 家，总数达 2.1 万家，解决就业 12.3 万人次 ，乡村旅游成为我市农民增收致富的重要渠道。

商贸服务业繁荣活跃。 中心城区城市经济日益繁荣，步步高、苏宁电器等一批名店纷纷落户饶城。 “ 万村千乡 ” 市场工程顺利推进，农家店已覆盖全市 100% 的县（市、区）、 90% 的乡镇、 80% 的行政村，商品配送率达 50% 以上。全面落实了汽车以旧换新、家电下乡等政策。

金融保险业健康发展。 南昌银行、招商银行在我市设立分支机构，上饶银行在市外 设立了 3 家分行，广丰、万年相继成立村镇银行。晶科能源在美国纽交所成功上市。全市金融机构年末本外币存款余额 979.7 亿元，较年初增加 195 亿元，增长 24.9% ；全市各项贷款余额 578 亿元，较年初增加 78 亿元，增长 15.6% 。保险业加快发展，全年提供各类风险保障 2 万亿元，民生保险、大地保险、永成保险落户信州区。

（三）内外互动，增强了改革开放的新活力

在推进经济社会发展中，始终坚持对内抓改革、抓全民创业，对外抓招商引资、抓外经外贸，为发展注入新动力、增添新活力。

不断深化改革。 在林权制度改革、国有工业企业改革和市县 两级政府机构改革顺利完成的基础上，去年又开展了七个系统国有企业改革，其中的五个系统国有企业改革基本到位，共安置职工 1.65 万人。医药卫生改革积极推进，并取得初步成效；国家基本药物制度在基层公立医疗机构实现全覆盖，基本药物销售率达到 71% ，药价平均降幅达到 30% ；农民看病用药费用下降 26.3% 。新一轮文化体制改革开始启动。

全力扩大开放。 充分发挥国家 加工贸易重点承接地、南昌海关驻上饶办事处、上饶无水港等开放平台作用，围绕重点产业、重点区域、重点企业，不断增强招商引资的主动性和实效性，开放型经济迈出了新步伐。全年实际利用外资 5 亿美元，同比增长 44.5% ，增幅列全省第一位；引进省外 5000 万元以上项目资金 188.2 亿元，同比增长 42% ；实现外贸出口 15.2 亿美元，同比增长 160% ，总量居全省第三位、增幅列全省第二位。

推进全民创业。 大 力实施 “ 创业带动就业 ” 战略，全民创业和服务水平不断提升，创业文化加快培育。全年发放小额贷款 4.8 亿元，净增个体工商户 28361 户，新增私营企业 2778 户，注册资本达到 406.6 亿元。

（四）项目带动，增强了基础设施的承载力

重大交通基础设施建设进展顺利。 完成国省道公路改造 158 公里、 农村公路建设 1234 公里、渡改桥 49 座，行政村基本实现通水泥路（油路）。合福高铁、杭南长高铁顺利推进。九景衢铁路、皖赣铁路扩能改造项目前期工作抓紧推进，今年将开工建设。上武高速、德昌高速加快推进；德上高速施工全面展开。三清山机场前期工作进展顺利。

电力能源快速发展。 上饶经济技术开发区 220 千伏输变电工程开工建设，新一轮农村电网改造已经启动。天然气主干管道全面开工铺设。

水利基础设施加快推进。 列入国家专项规划内的 147 座病险水库除险加固工程已基本完成；解决了 29.7 万农村人口饮水安全问题；灾后农田水利基本建设完成投资 14.2 亿元、土石方 1100 万方；伦潭水利枢纽工程顺利推进。

信息化建设成效良好。 通讯业快速发展，全市固定电话用户达到 86.6 万户，移动电话用户达到 286 万户， 3G 用户达到 7.1 万户，互联网用户达到 22.8 万户。电子政务加快推进，网上办公逐步开展， “ 数字上饶 ” 建设初见成效。

（五）民生牵动，增强了构建和谐的保障力

贯彻落实 “ 以人为本 ” 方针，把构建和谐平安上饶作为政府工作的重中之重，重民生、解民忧、保民安，促进了社会事业的全面进步。

民生保障持续增强。 全市拨付各项民生保障支出 42.3 亿元， 65 项民生实事全面落实。就业工作成效显著， 全年城镇新增就业人数 10.5 万人， “ 零就业 ” 家庭就业安置率达到 100% ， “4050” 人员就业人数 9016 人，新增转移农村劳动力 14.4 万人；安置近 8000 名残疾人就业。社会保障更加有力，全市城镇职工养老保险参保人数达到 45 万人 ；婺源县、余干县、信州区被列为全国新型农村养老保险试点。医疗保险惠及更多百姓，新农合农民参保人数达到 529 万人，参合率达到 97.6% ；城镇居民、城镇职工基本医疗参保缴费人数达到 138 万人，覆盖率达到 98.8% 。低收入群众得到有效保障，城乡低保人数达到 37.5 万人，发放低保金 5 亿元，实现应保尽保。助学力度进一步加大，资助家庭经济困难的普通高中学生、考入大学学生、中等职业教育学生达到 4 万人。 保障性住房建设进度加快，廉租住房全面开工，已完成 3389 套、 17 万平方米；以信州区为主体的棚户区改造已拆迁 87.6 万平方米。农村危房改造试点进展顺利，灾后重建竣工率达到 97% 。城市居民人均住房面积达到 30.5 平方米；农民人均住房面积 37.7 平方米。扶贫开发工作取得新成绩，完成了 5970 名贫困群众移民搬迁任务。

社会事业全面进步。 优先发展教育事业，九年制义务教育巩固提高，德兴市在全省率先实施高中阶段免费教育；全市投入 4.9 亿元，新（改）建、维修校舍面积近 50 万平方米，办学条件明显改善；高考再创佳绩，录取率达 83.2% 。强化公共卫生服务和管理， “ 九项基本 ” 和 “ 五项重大 ” 公共卫生服务项目进展顺利，市、县、乡、村四级卫生监督、医疗卫生服务网络基本形成；手足口病、甲型 H1N1 流感等 重大传染病得到有效防控；血防综合治理项目扎实推进，信州区通过省级传播阻断验收； “ 光明 · 微笑 ” 工程进入常态化管理；全面开展儿童白血病和先天性心脏病筛查，已免费救治白血病儿童 26 名。科技创新取得突破，申报国家级光伏、光学高新技术产业化基地取得成功，百勤异 VC 钠有限公司成立博士后工作站。 文体活动丰富多彩。继央视 “ 星光大道 ” 、 “ 心连心 ” 、 “ 欢乐中国行 ” 、 “ 开心辞典 ” 走进上饶后，再度携手央视举办 “ 爱国歌曲大家唱 ” 大型演出；组织实施 “ 高雅艺术进上饶 ” 演出 31 场，成功举办第二届农民艺术节和环鄱阳湖国际自行车大赛；新建乡镇综合文化站 88 个，新增农家书屋 558 个，完成村村通广播电视工程 485 个；组团参加省第十三届运动会并取得佳绩。高度重视计划生育工作，全年人口自然增长率 7.51‰ ，出生人口性别比 116.3 ，下降 3.5 个单位值，市人口计生委荣获全国人口计生工作先进集体称号。深入开展国防教育，部队和民兵预备役建设得到加强，驻饶解放军、武警消防官兵、民兵预备役为夺取抗洪抢险斗争伟大胜利作出了突出贡献。统计审计、新闻出版、外事侨务、涉台事务、气象水文、防震减灾、人民防空、妇女儿童、地方志、红十字、残疾人、老龄、档案、保密、慈善工作等各项事业都取得新成绩。

社会整体安全稳定 。安全生产形势进一步好转，全市事故起数同比下降 1.3% ，死亡人数同比下降 12.3% 。信访工作取得成效，赴京非正常访较上年下降 30.5% ，确保了世博会、中博会、亚运会等重大活动、重要节点的社会稳定。加强社会治安综合治理，扎实开展严打整治斗争，始终保持对犯罪活动的高压态势，确保了社会治安状况的全面稳定。全市刑事 “ 八类案件 ” 发案总数同比下降 14.5% ，公众安全感指数达到 95.6% ，较上年同期提高 1.2 个百分点。

生态建设扎实推进。 造林绿化 “ 一大四小 ” 工程建设连续两年被评为全省综合先进设区市。全市建有省级以上森林公园 27 处，其中国家级 8 处，森林公园数量占全省五分之一。完成规模化养殖场大中型沼气工程 39 个，居全省第一；新增户用沼气 1.6 万户、总量达 15.7 万户。强化环境监测，否决了 42 个有污染隐患的项目， 10 个县（市）污水处理厂全部投入运行。继续加大节能减排力度，全面完成省政府下达的目标。市内两大河流监测断面水质达标率 100% ，城市空气质量达到二级以上标准。上饶经济技术开发区和横峰工业园区省级生态园区试点建设通过验收，新增广丰、万年、婺源、余干 4 个省级生态园区建设试点。

（六）效能推动，增强了各级政府的执行力

一年来，各级政府以开展 “ 创业服务年 ” 活动为契机，进一步转变工作作风，不断提升机关效能，自身建设迈出新步伐。自觉接受人大法律监督、工作监督和政协民主监督及社会监督。广泛听取各民主党派、工商联、无党派人士以及各人民团体的意见和建议，充分发挥社团、行业协会和中介组织的积极作用。认真办理市人大代表建议 108 件、市政协委员提案 196 件，满意率达 96.9% 。深入开展 “ 创业服务年 ” 活动，被评为全省先进单位。进一步深化行政审批制度改革，严格执行了 “ 三重一大 ” 事项决策制度，扎实推进行政审批职能向一个科室集中、办理服务向一个窗口集中，网上审批和电子监察系统初步建成，行政审批和公共资源交易归并到位。进一步加强市长热线工作，全年共接听、办理市民电话 3 万余个，群众满意率达到 92% 以上。进一步加强应急管理工作，应对突发公共事件管理体制机制逐步建立，社会管理和公共服务职能不断加强。深入开展工程建设领域突出问题专项整治，严肃查处一批违法违规案件， 廉政建设取得积极进展。

各位代表，发展历程令人难忘，发展成就令人鼓舞，发展势头令人振奋 。 取得的成就来之不易，积累的经验弥足珍贵。这些成绩的取得，是我们坚决贯彻落实科学发展观的结果，是省委、省政府和市委统揽全局、正确领导的结果，是市人大、市政协和社会各界有效监督、鼎力支持的结果，是全市人民和衷共济、拼搏进取的结果。在此，我谨代表市人民政府，向全市广大工人、农民、知识分子、各级干部和离退休老同志，向各民主党派、工商联、无党派和社会各界人士，向驻饶部队、武警官兵和公安干警，向广大海内外投资者和所有关心支持上饶经济建设、社会发展的同志们、朋友们，表示衷心的感谢，并致以崇高的敬意！

在肯定成绩的同时，我们也清醒地认识到：尽管近年来我们发展速度加快，经济实力不断壮大，但总量偏小、人均值偏低的状况仍未得到根本改变；尽管近年来我市新型工业化、新型城镇化进程加快，对经济发展的拉动力不断增强，但与发达地区的差距仍然较大；尽管农业综合生产能力逐年提高，农民收入逐年增加，但农业抵御自然灾害的能力仍然比较薄弱，农民稳定增收的渠道还不够宽；尽管近年来民生投入大幅增加，广大群众得到了更多实惠，但就业和保障的压力仍然较大，部分群众生活还比较困难；尽管城市建设步伐加快，硬件设施明显改善，但城市管理中还存在一些亟待解决的问题，管理水平还有待进一步提高。对这些问题，我们将高度重视，认真研究，切实加以解决。

二、 “ 十二五 ” 时期的奋斗目标

各位代表， “ 十二五 ” 时期是我市推进鄱阳湖生态经济区建设、加快转变经济发展方式、为建设区域中心城市打下决定性基础的关键五年。未来五年，我们更加大有可为。一方面，宏观环境有利于我们加快发展。世界经济结构已进入调整期，创新和产业转型处于孕育期。中央将继续实行积极的财政政策和稳健的货币政策，我们既面临承接国际和沿海产业转移的大好机遇，也更有条件争取到国家更多的政策、资金和项目扶持。另一方面， “ 十一五 ” 平稳较快发展为今后的发展奠定了坚实基础。我市地处四省结合部，是江西对接长三角的桥头堡。随着打造现代交通综合枢纽步伐的加快，上饶的区位优势将更加凸显。因此，在未来五年，我们必须牢牢抓住机遇，将上饶放在全国、全省和区域发展大局中去思考、去谋划、去定位，进一步增强责任感和紧迫感，因势利导、顺势而为、乘势而上。

市委二届十一次全会提出了 《中共上饶市委关于制定国民经济和社会发展第十二个五年规划的建议》，明确了 未来五年的工作重点，即： 强力主攻 “ 三区 ” ，积极谋划建设大交通、大产业、大城市，致力于推进 “ 两个根本改变 ” ， 从而实现富民、强县、兴市的目标。 市政府 按照这个重点和 “ 抓好六个发展、实现六个更大突破 ” 的总体要求， 制定了《上饶市国民经济和社会发展第十二个五年规划纲要（草案）》，提交本次大会审议。

《纲要（草案）》提出了未来五年全市 经济社会发展总的 指导思想，即： 高举中国特色社会主义伟大旗帜，以邓小平理论和 “ 三个代表 ” 重要思想为指导，全面贯彻落实科学发展观，以科学发展为主题，以加快转变经济发展方式为主线，围绕 “ 建设四省交界区域中心城市 ” 的目标，打造 “ 一个枢纽 ” 、建设 “ 四个基地 ” ，强力主攻园区、城区和景区，加快推进鄱阳湖生态经济区、海西经济区建设，全力打造新兴工业强市、现代农业强市、全国旅游强市、居民最具幸福感城市，全力推动上饶科学发展、进位赶超、绿色崛起。

经济和社会发展预期目标建议为 ： 力争到 2015 年，全市生产总值年均增长 14% 、达到 2000 亿元；财政总收入年均增长 18% 、达到 260 亿元。与此同时，经济结构明显优化，城镇化水平快速提高，人民生活更加富裕，社会事业更加 繁荣，生态环境更加优良。

提出生产总值年均增长 14% 的目标，是在全面分析 “ 十二五 ” 时期发展条件和机遇的基础上，经过多方面科学测算确定的。

从三大需求拉动看： 预计 “ 十二五 ” 时期，固定资产投资累计将超过 8000 亿元，年均增长 20% ，年均拉动生产总值增长 10 个百分点；预计社会消费品零售总额可实现年均增长 16% ， 2015 年达到 690 亿元，年均拉动生产总值增长 3.5 个百分点；预计出口年均增长 20% ， 2015 年达到 38 亿美元，年均拉动生产总值增长 0.5 个百分点。 从三次产业发展看： 预计第一产业增加值年均增长 5% ， 2015 年可以达到 200 亿元，年均拉动生产总值增长 0.8 个百分点；预计第二产业增加值年均增长 19% ， 2015 年可以达到 1200 亿元，年均拉动生产总值增长 10 个百分点；预计第三产业增加值年均增长 13% ， 2015 年可以达到 600 亿元，年均拉动生产总值增长 3.2 个百分点。 从社会发展需要看： 年均增长 14 % 的安排，既考虑了要尽快缩小与全国、全省差距就必须加快发展的内在要求，又体现了加快转变经济发展方式、扩大就业、改善民生、维护社会稳定等现实需要；既体现了 “ 跳起来摘桃子 ” ，又是经过努力可以实现的。

三、 2011 年的主要任务

2011 年是中国共产党建党 90 周年，也是实施 “ 十二五 ” 规划的开局之年。做好今年的工作，意义重大，影响深远。按照市委二届十一次全会精神，今年全市经济社会发展预期目标建议为： 生产总值突破 1000 亿元，增长 13% 以上；财政总收入 136 亿元，增长 20% ；全部工业增加值 430 亿元，增长 19% ；固定资产投资 1038 亿元，增长 25% ；社会消费品零售总额 390 亿元，增长 18% ；实际利用外资 5.5 亿美元，增长 10% ；外贸出口 18.3 亿美元，增长 20% ；引进省外单项投资 5000 万元以上项目资金 216 亿元，增长 15% ；城镇居民人均可支配收入 17300 元，农民人均纯收入 5900 元，分别增长 12% ；城镇化率比上年提高 2 个百分点；居民消费价格总指数上涨控制在 4% 以内；人口自然增长率控制在 8‰ 以内；城镇登记失业率控制在 4.5% 以内；单位生产总值能耗、化学需氧量、二氧化硫、二氧化碳、氨氮化合物、氮氧化合物排放均控制在省政府下达的指标以内。

围绕上述目标任务，我们将着力抓好以下六个方面的重点工作：

　　 （一）狠抓项目，夯实基础强后劲

坚持以大项目带动大投入，以大投入带动大发展，继续增强固定资产投资对经济发展的拉动力。

扎实推进工业项目建设。 力争使工业项目投资突破 500 亿元，占固定资产投资总额的比重达到 50% 以上。继续推进晶科能源 3000 兆瓦光伏一体化， 和丰铜业 10 万吨电解铜，昂大公司 1000 万只铝合金汽车轮毂，黑马科技公司 1828 万包水晶钻石，韦伯尔铝业 6 万吨再生铝合金锭， 伟克铜材扩建年产 3 万吨铜管，江中余干分公司药品、卫生杀毒湿巾生产，源鑫电器公司电器陶瓷生产，昌硕纺织 2000 万米 户外休闲用品专用面料，江铜银山矿日采选 5000 吨铜精矿技改等重大项目。开工建设 江钨集团年产 2 万吨钴镍新材料， 鑫顺光伏年产 800 吨单晶硅棒及 300 兆瓦太阳能电池组件，江西中呈光电柔性太阳能电池等 项目。

加快推进重大基础设施建设。 全力配合合福高铁、杭南长高铁建设，推进上饶高铁客运站、九景衢铁路建设，加快皖赣铁路改造步伐；加快德上高速公路建设，确保上武高速、德昌高速年内竣工通车，开工建设三清山机场；加快 500 千伏东部环网输电线路建设，抓紧鄱阳湖核电、黄金埠电厂二期、上饶火电厂前期工作，配合抓好天然气入饶项目西气东输二线工程，推进宁德港上饶码头项目，加快启动鄱阳港务区建设。

大力推进农业基础设施建设。 深入开展 “ 农田水利建设年 ” 活动，抓紧实施堤防、灌渠、泵站及陂坝除险加固和更新改造，加快推进小（二）型以上水库除险和山洪地质灾害预警系统建设，进一步提高农田防洪排涝标准，实现新增、恢复和改善灌溉面积 25 万亩。基本完成伦潭水利枢纽工程建设。力争农业综合开发改造中低产田 6.6 万亩。

积极推进信息化建设。 建设第三代移动通信网络和无线城市，扎实推进 “ 数字上饶 ” 建设，优化网络布局，提高信息化网络的覆盖率、利用率。加快网络与信息安全保障体系建设，提升信息安全保障水平。

（二）抢抓机遇，扬优展势促转型

坚持以鄱阳湖生态经济区建设为契机，加快转变经济发展方式，不断提高产业发展的质量和效益。

1 、积极推进农业现代化。 牢牢抓住中央高度重视农业、不断加大 “ 三农 ” 投入力度的重大机遇，以农业增效、农民增收为核心，进一步加快现代农业发展，力争实现农业增加值 165 亿元，粮食总产稳定在 300 万吨以上。

提升农业科技贡献水平。 突出绿色生态优势，围绕粮食、畜牧、水产、果蔬茶、林产业等五大重点产业，深入开展粮食、果蔬茶高产创建活动，大力推进畜禽标准化和水产健康养殖示范基地建设，健全农技推广体系，提高农业机械化程度，加快建设一批示范点、示范片、示范带、示范区。

提升农业产业化经营水平。 以发展农产品加工业为重点，一手抓扶持，一手抓引进，培育壮大一批 市场前景好、发展潜力大、带动能力强的 农业龙头企业。加快万年生猪冷链物流项目建设。支持各类农民专业合作组织规范发展，鼓励企业开展 “ 农超对接 ” ，办好农业产业招商暨农产品展销会。

提升农业保障服务水平。 高度重视防汛抗旱、森林防火、动物防疫、农资防假工作，加强农产品市场流通、质量检验检测和气象防灾减灾体系建设，巩固和强化农业安全保障能力。完善农村 土地流转机制，积极发展适度规模经营。全面落实 强农 惠农政策，积极开展各类政策性农业保险，健全农业风险保障体系。

2 、加快新型工业化进程。 牢牢抓住国家新兴产业发展迅速、转型步伐不断加快的重大机遇，大力 实施工业三年强攻计划， 力争全市规模以上工业增加值达到 300 亿元、主营业务收入达到 1350 亿元、利税总额达到 115 亿元，分别增长 22% 、 25% 和 20% 。

增强园区集聚能力。 进一步完善工业园区道路、水电、通讯、污水处理等公共设施建设，加快土地整理和资源整合，着力提高园区的土地利用率、资源使用率、企业集聚率、单位面积产出率。加快建设广丰黑滑石产业基地， 积极推动上饶经济技术开发区特色产业基地建设。力争全市工业园区主营业务收入达到 1370 亿元，增长 25% ；其中上饶经济技术开发区主营业务收入达到 400 亿元，万年、铅山工业园区主营业务收入突破 100 亿元，全市百亿元园区达到 6 个，力争其它工业园区主营业务收入突破 50 亿元。

增强 产业支撑能力。 加快利用高新技术、先进适用技术嫁接、改造、提升四大主导产业的步伐。继续把新能源产业作为重点培育的战略性新兴产业。积极谋划新材料产业发展，把新材料产业培育成为我市又一战略性新兴产业。力争四大主导产业主营业务收入突破 1000 亿元，实现增加值 220 亿元，增长 25% ，其中新能源产业实现增加值 60 亿元。

增强龙头企业领跑能力。 抓好国家和省扶持企业发展的一系列政策措施的贯彻落实，对发展前景好、支撑带动力强的现有重点企业和重点技改项目，优先安排用地计划，优先提供金融服务，让优秀企业领先快跑，做大做强。鼓励企业围绕提高 “ 四率 ” 水平，加大研发投入，增强核心竞争力。高度重视中小企业发展，着力抓好中小企业孵化基地建设，支持中小企业走 “ 小而专、小而精、小而优 ” 之路。力争新增主营业务收入 10 亿元以上企业 5 户、 20 亿元企业 2 户，力争晶科能源主营业务收入突破 100 亿元，实现上饶百亿元工业企业零的突破。

3 、提高服务业发展水平。 牢牢抓住国家继续实施 “ 扩内需、促消费 ” 政策的机遇，充分利用我市丰富的旅游资源和独特的区位优势，促进现代服务业不断繁荣。

加快发展旅游产业。 围绕建设全国旅游强市的目标，继续做好三清山创 5A 级景区和神农源、鄱阳湖国家湿地公园、大茅山申报国家级风景名胜区工作；加快婺源、龟峰申报国家 5A 级景区和铜钹山、灵山、闽浙皖赣革命根据地旧址、葛仙山、神农源等申报国家 4A 级景区工作。进一步完善三清山、婺源、龟峰、鄱阳湖湿地公园等景区基础设施，建设德兴矿山公园。积极融入鄱阳湖生态经济区、长三角和海西经济区大旅游圈，高起点、高标准打造旅游精品线路。加快建设十大旅游名镇，大力发展乡村特色旅游，形成新的旅游消费热点，促进旅游产业转型升级。 力争 新增农家乐 5000 家，接待国内外游客 2400 万人次，实现旅游总收入 170 亿元，分别增长 20% 。

大力发展商贸物流业。 实施标准化市场示范工程，改造提升一批农贸市场，加快构建农村现代流通服务网络，不断完善城乡市场流通体系。继续引进名店、名企落户上饶，积极培育连锁化经营的大型流通企业。 依托高速公路、高铁路网等交通优势，高起点规划一批大型物流基地。加快引进民间资本，积极发展民营物流企业。力 争培育 1-2 个销售收入过 20 亿元的商贸物流企业。同时，加强市场监管，强化食品药品安全，确保人民群众饮食用药安全。抓好物价调控，通过提高供给保障能力、发放价格临时补贴、严肃查处各类价格违法行为等措施稳定市场物价，切实保障群众基本生活。

积极发展金融保险业 。 继续支持各类金融机构落户我市。支持上饶银行在市外发展网点，并实现市内县（市、区）支行全覆盖。进一步促进政银企合作制度化、常态化，切实提高签约项目履约率。健全企业融资担保体系，推进信用体系建设，构建优良金融生态环境，力争全市金融机构本外币存款余额新增 200 亿元，贷款余额新增 100 亿元。积极发展各类商业保险，进一步发挥保险保障功能。有序推进企业股份制改造和上市工作，力争培育 1-2 家企业上市。

4 、强化节能减排。 围绕 “ 走绿色崛起之路、建和谐生态之城 ” 的目标，坚持低碳、低耗、低排、低污 “ 四低 ” 并重，着力推进 生态文明建设 。巩固 上饶经济技术开发区、横峰工业园区省级生态园区建设成果，加快广丰、万年、婺源、余干 4 个工业园区创建省级生态园区试点步伐，新增 1-2 个县（市）工业园区进入省级生态园区试点行列。加快县城和重点镇生活垃圾处理设施建设，提高 10 个县（市）污水处理厂运行效率；高度重视工业园区污水处理设施建设。进一步加强环境保护和污染治理，推进重点区域、重点行业和重点流域环境整 治，全面完成省政府下达的节能减排目标任务。加强信江、乐安河源头保护，强化饮用水源地保护。坚持依法用地和节约用地。深入推进造林绿化 “ 一大四小 ” 工程，完成 45 万亩造林任务。启动森林城乡 “ 十项创建 ” 活动，创建森林乡镇 60 个、森林村庄 200 个。

（三）统筹城乡， “ 双轮驱动 ” 助提升

坚持把推进新型城镇化作为主攻方向，加快构建布局合理、结构优化、特色鲜明的现代城镇体系 。

提升中心城区功能。 优先改善市民生活设施， 开工建设大坳引水工程，加快汪家园棚户区、长塘棚户区、槠溪河东岸危旧房、铁路新村改造步伐，推进一批幼儿园、小学、社区医院、老年公寓、文体设施、菜市场、停车场、小游园、公厕等与市民日常生活紧密相关的设施建设。切实加大保障性住房建设力度，科学调控商住用地供应，保持房地产市场健康发展。 建立健全住房公积金制度。 突出 建设城市基础设施， 新建和续建天佑大道、龟峰大道、滨江路、龙潭路、茶圣路等一批道路工程；新建龟峰大桥，改造龙潭大桥、紫阳大桥等一批桥梁工程。加快提升公共服务设施，启动三江口城市综合体、老体育中心综合体、老火车站片区综合体、上饶市旅游集散中心等一批重点工程。注重完善绿化景观设施，以创建国家园林城市为载体，将三清大道、凤凰大道、迎宾大道改造提升为林荫大道；加快新老城区、新建小区、新修道路的绿化景观建设，推进上饶市创业文化公园、辛弃疾公园、陆羽公园、茅家岭公园、槠溪河湿地公园、花卉博览园、三江水系改造等工程，完成云碧峰森林公园入口处改造。

提高城市管理效能。 进一步理顺中心城区城市管理体制，切实加强城市管理 。以文明创建为载体，进一步加强中心城区公交、出租行业管理和公共交通秩序整治，下大力气解决一些重点区域、重点地段 “ 行车难、乘车难 ” 问题；进一步加强火车、汽车客运站点等人员密集场所综合整治，维护文明有序的公共秩序；进一步加快里弄小巷、社区公共设施的配套完善，让市民出行更快捷、生活更方便、环境更舒适；深入开展 “ 美化饶城、文明有我 ” 活动，大力倡导文明新风，切实增强市民的城市归属感和自豪感。

加快县城和中心镇建设。 按照县城建设 “ 十个一 ” 和示范镇建设 “ 八个一 ” 的要求，不断加大县城和示范镇建设力度，力争鄱阳、广丰县城人口达到 25 万人左右、建成区面积达到 25 平方公里以上，余干县城人口达到 20 万人、建成区面积达到 20 平方公里以上；力争其它县城人口达到 10 万人左右、建成区面积达到 10 平方公里以上。全面完成省、市示范镇三年发展规划的编制和总规修编工作，加快建设一批城镇道路基础设施、学校、医院等社会事业项目和集贸市场、自来水等民生工程项目，积极营造宜居宜业城镇环境。

深入推进新农村建设。 突出湖区、景区、郊区 “ 三区 ” 特色，扎实推进 1300 个新农村村点建设。按照 “ 减量化、资源化、无害化 ” 要求，推进 5012 个农村清洁工程试点建设，巩固提升新农村建设成效。

加快发展县域经济。 充分调动和发挥县（市、区）的积极性、主动性和创造性，依托县域优势，优化产业布局，推动县域经济快速发展。力争广丰县、德兴市财政总收入突破 20 亿元，信州区、上饶县、玉山县、铅山县财政总收入突破 10 亿元，其它县财政总收入都突破 5 亿元。

（四）改革开放，开拓创新增活力

坚持改革开放不动摇，通过深化改革破解发展难题，在扩大开放中赢得发展机遇。

积极推进各项改革。 稳妥推进农垦企业改革，基本完成七个系统国有企业改革任务。积极推进乡镇机构改革， 稳步推进医改工作。实施国家基本药物制度，探索公立医院改革，努力完成国家基本和重大公 共卫生服务项目，促进基本公共卫生服务逐步均等化。 深化文化体制改革，推动经营性文化单位转企改制。

全方位推进招商引资。 进一步落实 “ 招商引资十项措施 ” ，继续围绕重点产业、重点区域、重点企业，加大招商引资力度，不断引进大项目、大企业。特别是在跟进、强壮主导产业上下功夫， 力争 重点产业招商实际进资突破 150 亿元。

大力推进外经外贸。 加快上饶铁路口岸作业区建设，开通上饶至上海的海铁联运，加速上饶至宁波 “ 五定班列 ” 进程。加紧做好国家级上饶出口加工区申报和南昌海关驻上饶办事处升格工作。支持企业 “ 走出去 ” ，壮大外贸出口主体，做大外贸出口。

深入推进全民创业。 一如既往地鼓励、支持和引导民营经济发展，让创业者在法律上有保障、政策上有支持、舆论上有鼓励、社会上有声誉，努力营造 “ 政府鼓励创业、能人带动创业、百姓自主创业 ” 的浓厚氛围，充分释放群众的创业潜能。 继续发挥工商联、行业协会、商会等组织的桥梁纽带作用，切实保护好创业者的合法权益，促进民营经济健康发展。

（五）全面推进，以人为本兴和谐

坚持优先解决与人民群众切身利益密切相关的问题，维护社会稳定，努力构建和谐社会。

1 、继续实施好民生工程。 坚持把保障和改善民生作为政府工作的重中之重，围绕省政府确定的八个方面的民生实事，加大力度，加快推进，努力让上饶的百姓生活得更加舒心、更加健康、更加幸福。

在就业和创业方面： 实施更加积极的就业政策，确保新增城镇就业 5.1 万人，新增转移农村劳动力 7.5 万人。以用工需求为导向，加强培训与就业的对接，完成劳动力转移培训 6 万人。实现 “ 零就业 ” 家庭就业安置率达到 100% ，为残疾人购买公益性岗位 390 个。充分发挥小额担保贷款扶持创业、促进就业的作用，新增小额贷款 3.1 亿元。

在社会保险方面： 扩大城乡社会保障覆盖面，城镇职工基本养老保险参保人数达到 46 万人，基本养老保险基金征缴总额达到 11.2 亿元；妥善解决未参保城镇小集体企业职工参加城镇职工基本养老保险问题；做好婺源县、信州区、余干县新农保试点工作，新农保参保人数达到 72 万人；将符合条件的被征地农民纳入基本生活保障范围，人数达到 4.5 万人；失业保险基金征缴达到 4850 万元。提高企业退休人员基本养老金水平，提高居委会干部和 “ 三院 ” 工作人员补贴。

在医疗保险方面： 进一步完善医疗保障 “ 三张网 ” ，城镇基本医疗保险参保人数达到 127.8 万人，其中城镇职工参保人数达到 53.8 万人，城镇居民参保人数达到 74 万人，城镇职工和居民基本医疗保险统筹基金年度使用率达到 90 ％；工伤保险参保人数达到 30.6 万人，其中农民工参保人数达到 7.1 万人；生育保险参保人数达到 17.6 万人；提高新农合保障水平，统筹基金使用率达到 85 ％以上，参合农民住院一次报账率达到 90 ％以上；免费提供价值 310 万元的社区公共卫生服务卡，农村居民规范化健康档案建档率达到 30% ，残疾人康复 2 万人；继续实施大病救助制度，对全市所有符合救治条件的儿童白血病患者实施免费救治。

在社会救助方面： 加大济困力度，将城市低保保障标准提高到 300 元以上，人均月补差标准提高到 200 元；农村低保保障标准提高到 130 元以上，人均月补差标准提高到 85 元；集中供养和分散供养的农村五保户年供养标准分别提高到 2400 元和 1560 元；城镇 “ 三无 ” 特困群众的月人均供养标准提高到 350 元。提高城镇大集体困难企业未参保退休职工、国有农林水困难企事业单位未参保退休职工、 20 世纪 60 年代精简退职老弱残职工等困难群众的月人均生活补助标准。奖励扶助农村计划生育家庭 6700 人、特别扶助 1050 人。

在教育、卫生方面： 继续实行义务教育阶段免除学杂费、免费提供教科书政策；进一步落实家庭困难学生资助政策；加快城镇新区教育园区建设，提高边远地区教师特殊津补贴，推进校舍安全工程建设。实行国家基本药物制度的乡镇卫生院达到 238 个、社区卫生服务机构达到 20 个。

在保障性住房建设方面： 新增廉租住房 3318 套，建设公共租赁住房 1554 套，完成城市棚户区改造 66.6 万平方米，为农村困难群众新建、重建住房 446 户、维修住房 227 户。

在扶贫开发方面： 完成 7249 名深山区、库区、地质灾害频发区贫困群众移民搬迁，财政安排 6832 万元实施整村推进扶贫规划。

在提高群众生产生活水平方面： 建设 农村公路 1280 公里，建设农村候车亭 250 个；新增农村户用沼气 1 万户，解决 25 万农民安全饮水问题；建设 880 个农家书屋，送戏下乡 900 场以上，建设村村通广播电视工程 300 个。

2 、协调发展各项社会事业。 深入实施教育优先发展战略，巩固提高义务教育，努力扩大优质高中资源 ；大力发展职业教育、高等教育和学前教育；加快上饶中学建设，确保今年秋季开学 。加强卫生能力建设，健全基层医疗服务体系； 抓紧重大传染病防控、鄱阳湖南岸片区血防综合建设试点工作，严格控制艾滋病、血吸虫病、结核病等重大传染病传播；加强基层卫生监督网络建设，提高卫生监督服务能力和水平； 推进中心城市公共医疗设施建设； 继续实施科技创新 “633611” 工程，加大科技投入，支持科技创新；加大人才建设力度，不断壮大 “ 高、精、尖 ” 人才队伍。 承办好第七届全国城市运动会女子足球赛事，广泛开展群众性体育活动。 推动文化设施建设，加强以图书馆、文化馆、博物馆、乡镇综合文化站为重点的公共文化服务体系建设 ，做好全市有线电视数字化整体转换工作；加快发展文化创意、现代传媒等文化产业，着力打造戏曲文化、红色文化、生态文化、稻作文化等品牌，不断提升文化软实力。加强国防教育，持续推进民兵预备役建设，努力争创“全国双拥模范城”。

坚持计划生育基本国策。 稳定低生育水平， 有效遏制出生人口性别比偏高现象，强化流动人口管理和服务，完善利益导向机制，实施优生促进工程，实现 “ 一升三降 ” 目标。

3 、切实维护社会安全稳定。抓好安全生产， 进一步落实安全生产责任制，完善安全生产监管体制，夯实基层监管基础，强化政府监管职责，明确企业主体责任，进一步加强重点行业、重点领域、重点企业的安全监管工作。深入开展专项整治活动，消除各类隐患，坚决杜绝各类重特大安全事故发生。积极推动应急救援体系建设，提高应对自然灾害、事故灾难、公共卫生事件、社会安全事件的快速反应能力。有效化解社会矛盾，坚持用群众工作统揽信访工作，健全和完善接访下访工作机制，畅通社情民意反映渠道，加大矛盾纠纷排查调处力度，构建人民调解、行政调解、司法调解相结合的 “ 大调解 ” 工作格局。妥善解决土地征用、房屋拆迁、企业改制、医患纠纷等引发的各类矛盾，最大限度地消除不稳定因素。加强社会治安综合治理，不断完善社会治安防控体系建设，严厉打击各类违法犯罪活动，有效降低可防性案件发案率，提高各类案件破案率；认真做好青少年的维权、预防违法犯罪、刑释解教人员安置帮教和社区矫正工作，为人民群众营造安居乐业的社会环境。

（六）改进作风，求真务实抓发展

坚持加强政府自身建设，不断提高政府系统执行力和服务水平，全面适应新形势和新任务的要求。

推进依法行政。 自觉接受市人大及其常委会的法律监督和工作监督，主动接受人民政协的民主监督，及时办理人大代表议案、建议和政协委员提案，不断提高办理质量和满意率。认真听取各民主党派、工商联、无党派人士和各人民团体的意见。加强监察、审计专门监督，高度重视新闻舆论和社会公众监督。不断加强政府法制工作，政府出台的规范性文件，件件都要经过法制审核；政府的每一项行政行为，事事都要经得起法律检验。提升行政执法队伍素质，提高行政执法水平，使每一个公务人员在执法过程中都能管得住自己、帮得了百姓，真正让老百姓看得起、信得过。

推进科学施政。 政府各项决策都要符合民主决策要求，建立健全领导、专家、群众相结合的决策机制，完善重大决策、重要事项的调查研究、公开咨询、听证公示制度，提高决策透明度和公众参与度。政府各项决策都要符合群众意愿、维护群众利益，把群众关心的小事当做大事来办，把群众关注的热点作为重点来抓。政府各项决策都要符合客观规律，坚持从实际出发，按客观规律办事，树立正确的政绩观；办事业既要尽力而为，也要量力而行；做项目要想好了再说、说好了再干、算好了再上。努力用实实在在的业绩，换取上饶实实在在的发展，带给群众实实在在的利益。

推进高效理政。 巩固扩大 “ 机关效能年 ” 和 “ 创业服务年 ” 活动成果，深入开展 “ 发展提升年 ” 活动，进一步提升行政效能。各级政府要力避 “ 文山会海 ” ，把精力用在谋发展上、把功夫下在抓落实上，力戒 “ 以会议贯彻会议、以文件落实文件 ” 的漂浮作风。要转变行政职能和服务方式，减少行政审批，简化办事程序，积极推进电子政务，推行网上审批和电子监察，实施重大产业项目审批 “ 绿色通道 ” ，实行公开办理、限时办结。要强化责任落实，坚持谁主管、谁负责，谁经办、谁负责，一级抓一级，人人责任明确，层层奖罚分明。

推进廉洁从政。 政府的各级领导要坚持以身作则管牢自己，带头遵守《廉政准则》，大行廉政之风，做到稳得住心神、经得住诱惑、守得住底线。坚持 “ 一岗双责 ” 管好队伍，加强对分管系统、分管部门以及工作人员的廉政教育，努力形成 “ 一要干事、二要干净 ” 的良好风气。坚持健全制度管住根本，推行风险岗位廉能管理制度，严肃查处各类违纪违法案件。加强对公共资源交易和招投标，以及工程建设等重点领域和关键岗位的审计、监察和监管。积极推进节约型机关建设，严格控制一般性支出，勤俭办一切事业。

各位代表，新的蓝图宏伟而壮丽，新的任务艰巨而光荣。我们对上饶的美好前景充满期望和信心，对建设美好的上饶充满激情和干劲。让我们更加紧密地团结在以胡锦涛同志为总书记的党中央周围，在中共上饶市委的坚强领导下，开拓创新、锐意进取，同心同德、扎实工作，为实现 “ 十二五 ” 规划宏伟目标、建设美丽富饶的新上饶而努力奋斗！
